# Understanding the dynamics of the *Seguro Popular de Salud* policy implementation in Mexico from a complex adaptive systems perspective

**DOI:** 10.1186/s13012-016-0439-x

**Published:** 2016-05-13

**Authors:** Gustavo Nigenda, Luz María González-Robledo, Clara Juárez-Ramírez, Taghreed Adam

**Affiliations:** 1 School of Medicine, Morelos State Autonomous University, Calle Leñeros esquina Iztaccíhuatl s/n Col. Volcanes, Cuernavaca Morelos, CP 62350 Mexico; 2 Center for Health Systems Research, National Institute of Public Health, Av. Universidad 655, Santa María A, Cuernavaca, CP 62100 Mexico; 3 Health Systems and Innovation, World Health Organization, 20 Avenue Appia, Geneva, Switzerland

**Keywords:** Complex adaptive systems, Universal health coverage, Stakeholder analysis, Causal loop diagram, Seguro Popular, Mexico

## Abstract

**Background:**

In 2003, Mexico’s *Seguro Popular de Salud* (SPS), was launched as an innovative financial mechanism implemented to channel new funds to provide health insurance to 50 million Mexicans and to reduce systemic financial inequities. The objective of this article is to understand the complexity and dynamics that contributed to the adaptation of the policy in the implementation stage, how these changes occurred, and why, from a complex and adaptive systems perspective.

**Methods:**

A complex adaptive systems (CAS) framework was used to carry out a secondary analysis of data obtained from four *SPS*’s implementation evaluations. We first identified key actors, their roles, incentives and power, and their responses to the policy and guidelines. We then developed a causal loop diagram to disentangle the feedback dynamics associated with the modifications of the policy implementation which we then analyzed using a CAS perspective.

**Results:**

Implementation variations were identified in seven core design features during the first 10 years of implementation period, and in each case, the *SPS*’s central coordination introduced modifications in response to the reactions of the different actors. We identified several CAS phenomena associated with these changes including phase transitions, network emergence, resistance to change, history dependence, and feedback loops.

**Conclusions:**

Our findings generate valuable lessons to policy implementation processes, especially those involving a monetary component, where the emergence of coping mechanisms and other CAS phenomena inevitably lead to modifications of policies and their interpretation by those who implement them. These include the difficulty of implementing strategies that aim to pool funds through solidarity among beneficiaries where the rich support the poor when there are no incentives for the rich to do so. Also, how resistance to change and history dependence can pose significant challenges to implementing changes, where the local actors use their significant power to oppose or modify these changes.

## Background

Health systems are complex adaptive systems characterized by the constant interaction and feedback between its constituting parts, leading to continuous changes and adaptation and often unexpected consequences [1, 2]. Complex adaptive systems frameworks have been increasingly used in recent years to understand the complex nature of introducing new policies and how they work and influence health systems [3–6], but applications of how this can be done in practice are still scarce, especially in low- and middle-income countries [5, 7–12]. This paper seeks to add to this body of knowledge and understanding by applying a complex adaptive systems (CAS) framework to the question of how do health systems actors respond to new policies, more specifically, how do they react to, adapt or change the path of policy implementation with respect to the way it was conceived. We used the implementation of the financial reform policy “*Seguro Popular de Salud*” (SPS) in Mexico as a case study to explore this question.

In 2003, Mexico’s public health system experienced a historical change in its financial structure. After decades of chronic underfunding, the Health Social Protection System (HSPS), and its financial component *Seguro Popular de Salud*, was introduced to channel new funds to provide a broad package of health services to around 50 million Mexicans who were not affiliated to any of the traditional social security institution [13], founded to provide health and other social services to formal industrial workers. By 2012, SPS managed to generate an unprecedented flow of financial resources equivalent to the triple of resources that the Ministry of Health (*SSA in Spanish*) accounted for in 2000 [14]. As a financial policy within the public system, SP defined its objectives to (a) improve the distribution of public financial health resources across the Mexican states, (b) reduce the inequities between those covered by social security schemes and the rest of the population, and (c) reduce out-of-pocket and catastrophic expenditure. The first two were clearly accomplished over the rolling out period [15, 16], but the third has only been partially accomplished. Literature shows that the effect of SPS has concentrated on the reduction of catastrophic expenditure [17, 18].

In addition, SPS was also conceived to produce improvements in the performance of the public system including increasing the number of the affiliated population and the distribution of non-financial resources (personnel, medicines, equipment) among the affiliated populations throughout the country [19–22]. Empirical evidence demonstrated improvement in patient satisfaction due to drug availability, reduced waiting times and better treatment for ambulatory and hospital care [13, 23, 24] and improved access to specific services such as screening for breast cancer, diagnosis and treatment of diabetes, and specific vaccines (e.g., measles) [13]. There are also indications of reduced inequities in access to health services, but the evidence is not yet conclusive. Important reduction in the use of private hospital services was also observed for new affiliated population under SPS [25–27].

Although SPS has been demonstrated to produce various relevant effects, not enough attention was given to the implementation process and its implications on the health systems and its overall goals. Therefore, while SPS has achieved several hard-to-reach objectives, it is still confronting enormous managerial challenges that could be hindering its ability to ensure that its ultimate goal is fulfilled—effective universal health coverage of all Mexicans, through equitable access to affordable and good quality health services with financial risk protection [13, 28]. After more than 10 years of its inception, the policy implementation process has endured important variations across the 32 states [13], but we still do not clearly understand the reasons behind these variations and how they may have affected SP capacity to accomplish systemic managerial goals.

### Health systems as complex adaptive systems

Health systems share the same characteristics of CAS [29]. Although CAS frameworks and methods have been widely used to explore and understand complexity in various disciplines such business, management, and education, its application in health is relatively recent [12, 30, 31]. In the past decade, several studies concerned with health systems interventions or strengthening indicated the importance of using a different perspective than the traditional linear approach that have been predominantly used in the health literature so far, but little guidance is available as to how to apply it [30, 32, 33]. The relevance of understanding and analyzing health systems problems using a CAS perspective stems from the following fundamental characteristics of CAS that also characterize health systems [2, 29, 34]. The interactions among their components are non-linear and constantly changing, their dynamics are unpredictable and sometimes counter-intuitive, and they are capable to self-organize, adapt, and learn from experience on a constant basis [35–37]. Therefore, this inherent interconnectedness and interdependence between health systems components, its actors, and the context in which they operate create a continuous process of feedback loops with unpredictable time lags between the cause and effects, collectively creating a “dynamic complexity” [38].

As described by Paina and Peters (2011), CAS framework provides a useful approach to analyze the behavior generated by complex systems and its actors to understand how the behavior was produced, what were the associated phenomena that occurred, and how they influenced the system and the implementation of policies [31]. Some of these CAS phenomena include *network emergence*, where new hubs emerge that use their collective power to influence the system in a way that is greater than the sum of their individual powers; *path dependence*, where states are not only influenced by the policy itself but also by the initial conditions in each of these states and decisions taken along the way; *phase transitions* where threshold effects occur creating a new status in the system “whether around the rapid adoption of a policy stalled for years, changes in social norms concerning health behaviors, or a new demand for health services”, and *feedback loops* where an input to the system generate a reaction or an outcome that feedbacks into the system as an input to generate new processes or effects [31].

All these phenomena are very relevant to our research question. Understanding how *Seguro Popular de Salud*’s policy implementation follows or differs from its operational guidelines will not only clarify how and why SPS’s implementation took the routes it chose during these 10 years but also illuminate the thinking and design of future policies. What causes these changes? Why implementation guidelines are so difficult to implement as conceived? All these questions require an analytical approach that embraces complexity and the adaptive nature of complex systems such as the health system.

The objective of this study is, therefore, to apply a complex adaptive systems perspective to understand (1) the role of key actors and the way their responses and power shaped the policy through its implementation; (2) how the system adapted and modified the implementation of the policy and; (3) the dynamics by which this happened. The answers to these questions offer a critical insight to potential revisions or redesign of the policy and/or development of an alternative one.

## Methods

We used a case study approach to analyze the implementation of a financing reform policy, the *Seguro Popular de Salud* in Mexico from a CAS perspective This approach was selected for the following reasons: (1) the case study permits to analyze in depth a contemporary phenomenon in its real context, especially when the limits between the phenomenon and its context are not evident; (2) it focuses on responding the “hows” and the “whys” of a scarcely studied phenomenon; (3) relevant lessons could be obtained from a complex situation, based on the overall understanding of such situation, and (4) the results of the study can contribute to yield proposals for the definition or redefinition of public policies and give way to new research proposals [39, 40].

### Data sources

A combination of secondary data sources were used to inform our analysis, the majority of which were from four consecutive evaluations of *Seguro Popular de Salud*’s implementation processes carried out between 2007 and 2012 and published elsewhere [19, 39–43]. The four evaluations interviewed a total of 515 decision-makers at the federal and states levels, 1031 doctors and nurses from primary care units, and 2485 beneficiaries, using structured and semi-structured interviews. These reports also contain data on the number of enrolled beneficiaries, management of financial resources, and the structural and legal status of the financial intermediary unit in the different states as well as key informant interviews with managerial and technical staff at national and state level around various implementation processes and associated challenges [22–24]. Three of the authors (GN, LMGR, and CJ) participated from the start in the planning and conduction of the four evaluations as well as in the field data collection. However, in each evaluation a team of 15 researchers were involved along the process. The evaluations were financed by the Federal Secretariat of Health and were carried out by the National Institute of Public Health of Mexico (NIPH).

Other sources of information include financial flows obtained from the National Commission; the SP conceptual, financial, and operational guidelines; and laws, guidelines, and other normative documents that support the SP operations including the 2007–2012 SP “White Book” [44].

### Applying a complex adaptive systems framework

Peters DH (2014) provided a comprehensive review of the relevant methods to address problems of complexity within health systems [12]. The methodological paper by Paina and Peters (2011) provide a detailed framework for how to explore and analyze complex interventions as they are scaled up using a complex adaptive systems perspective [31]. They provide descriptions of the different phenomena that may occur when systems change as a reaction to intervening as described above, with examples of how they can be explored and tested, which we applied in this study. More specifically, data from the four SPS evaluations were reanalysed to explicitly explore what changes occurred in the policy over the first decade if its implementation, how the change was triggered, by whom action was taken, and how this affected the policy and its intended goals. Whenever a CAS phenomena or an action (by the system or its actor) was detected, additional secondary data sources were sought to further explore or confirm the observation. This iterative process was visually depicted using a causal loop diagram as explained below to specifically look at relationships between triggers, actions, and change in the form of feedback loops. Causal loops are very good means to go through such process as was successfully used by Rwashana et al. 2014 [4], Varghese et al. 2014 [11], and Paina et al. 2014 [5].

### Analysis of data

First, a detailed analysis of the main actors, their incentives, role, power, and responses to the implementation of the policy, including the way each of them adapted or interpreted the policy at its different phases of development, was performed. Then, the various changes and adaptations in the policy’s implementation and the reasons and dynamics involved in these adaptations were extracted from the various data sources taking into account different variations to policy adaptations in various states or by various actors. Next, the study team, which constitutes multi-disciplinary Mexican and international researchers, used this information to interpret the changes in the implementation of the policy and the mechanisms by which they happened. This iterative process was guided by the development of a causal loop diagram depicting the original and modified operational framework and the feedback loops that evoked those changes. This stage of the analysis was instrumental for the next and final stage, which employed a CAS framework to explore the mechanisms by which the changes occurred and the associated CAS phenomena (see below).

### Causal loop diagram

The generated list of changes in the implementation of SPS and its link to the various actors (see Table 1) were used to develop a causal loop diagram to understand how the implementation of policy was carried out over time and what were the dynamics involved in this process. Causal loop diagrams provide a useful approach to illustrate complex systems characteristics such as dynamic relationships, non-linearity (e.g., in the form of delays in outcomes after an initial intervention or action), and feedback loops. They offer a means to understand, interpret, and discuss dynamic relationships using a common terminology and techniques [45]. Variables are usually labeled in neutral terms using positive and negative signs on the arrows that link variables to show the direction of influence of one variable on another. Feedback loops occur when arrows connect a variable to itself through one or a series of variables.

**Table 1:** Main actors, original and modified roles, and influence in the allocation and management of *Seguro Popular* financial resources

Actor	Objective/iIncentives	Role in the system	Power	Policy responses	Source
National Commission of the Health Social Protection Policy (considered in the guidelines and had the same role as originally intended)	Ensure that the policy achieves its desired goals	To manage financial resources and transfer them to the states Coordinate the operation Stewardship To evaluate the REPSS performance as well as the overall system. To support the accountability of funds	High but without legal capacity of sanctioning It can delay disbursement of funds, e.g., if financial reports are not received It cannot act if they detect mishandling of resources	-Adapted and modified the policy -Changed the definition of capitation from family to individual -Accepted the state’s 15 % financial share to be represented by previous investments -Introduced a cap on spending on medicines (30 %) and hiring (40 %) -Families belonging to III and IV deciles were exempted from pre-payment	[24, 42]
State government treasury (not explicitly mentioned in the original guidelines but having a role)	Provide a mechanism for auditing the flow of funds	Receive funds from the National Commission and register them in the state’s financial system	High because of legal capacity to handle finances and sanctioning	-Kept funds as much as they can to obtain bank interests	[22, 23]
State Ministry of Health (considered in the guidelines but its role changed)	Provide health services through its public network	Receive funds and allocate according to capitation	High, e.g., in terms of fund allocation according to their priorities and network since it substituted the original role of REPSS	-Kept REPSS inside its structure to keep hold of federal financial resources -Did not implement capitation but maintained historical budget due to lack of information and managerial capacity -Initially issued short staff contracts to reduce costs but under pressure had to extend contracts -Limited private sector purchasing of services	[22, 33, 43]
State Health Social Protection Regime (REPSS) (considered in the guidelines but changed its role)	*Original*: Purchasing of services from public and private in an equitable and efficient way *Revised (except in one state):* ensure the highest number of affiliates registered and reported to bring more funds to the state	*Original*: financial intermediary Affiliate new users Protect users’ rights Management of financial resources Purchasing of services, Accountable to state and federal authorities *Revised (except in one state):* Administering the funds from affiliating new people into the plan. Transfer this information to the National Commission To participate in the allocation of resources to public health units	Low power or influence in allocation of funds Their role shrunk over time by being absorbed by the state MOH Although they existed under the MOH, they became increasingly passive	-Increased number of affiliated families, e.g., by re-interpreting the guidelines to identify single member families, to increase the funds allocated to the state	[43, 44]
National Workers Union (not considered in the guidelines but acquired an active role)	Represent the interests of unionized workers towards the employer	Negotiate the regularization of contracts. Monitor the process of regularization	High: Every regularized worker pays a 2 % fee of the value of the contract to receive protection from the union (contracts consumes between 40–60 % of the system’s total SP funds)	-Became active in the regularization of contracts process by negotiating with top federal players	[23, 24]
Contracted workers (not considered in the guidelines but having active role)	Obtain contracts to provide services	Participate in the delivery of services to the SP affiliated population	Low: they did not put pressure to obtain better contracts	Became active in the regularization of contracts process by accepting new contracts negotiated by the union	[23]
Pharmaceutical retailers (Not considered in the guidelines but having an active role)	Participate in bids and sell their products	Negotiate the selling price of medicines with each state	High: there are limited number of retailers and they lobby to agree on medicine pricing levels	-Depending on the state, retailers negotiated highly profitable contracts -Used their corporative and marketizing capacity to sell their products and to agree on drug prices used in bids	[23, 45]
Pharmaceutical distributors (NEW) (not considered in the guidelines but acquired an active role)	Win the bid for distributing drugs within the state	Negotiate to win the bid	Low—as there is more competition	-Used different marketing strategies to win distribution bids and to convince the states that they could reduce allocation times despite the cost involved.	[23]
State health bureaucracy (considered in the guidelines and had an active role)	As possible: -Use funds to cover its needs -Continue to function as before	Management of *Seguro Popular* funds at different levels and activities	High—in terms of flexibility to manage and spend funds	As initially no sanction system existed (before auditing started in 2009), some: 1. Bought medicines at high prices 2. Bought non-authorized goods 3. Contracted health workers without demonstrated competence	[23, 24]
Health units (providers) (considered in the guidelines but had a passive role)	Provide health services according to population needs and their capacity	Receive resources and provide services to the affiliated population	No power as they do not receive any funds directly	-No incentive to change status quo—business as usual -Complained about not being heard or participate actively in the allocation process or decision—e.g., responding to the health challenges they face	[22, 23, 43]
Beneficiaries affiliated to *Seguro Popular* (considered in the guidelines and acquired an active role)	*Original:* Had the right to choose providers *Current:* In practice, cannot exert that right as they have to deal with limited number of providers (mostly public sector)	Recipients of health services contained in the package of benefits	Low but increasing—e.g., if they organize themselves to exert more pressure	-As they received information about their rights, they increasingly became more vocal in obtaining better services and medicines	[24, 44]

There are two types of feedback loops. Balancing loops, also called neutralizing loops, can be seen when a resulting outcome was intending to neutralize an action or a policy to bring the system back to the desired state. Reinforcing loops depict a vicious circle that occurs when an action creates an exponential outcome that keeps increasing overtime, being reinforced by the variable that caused it, until a break in the cycle happens. Reinforcing loops can be positive (desired) or negative (undesired). A reinforcing loop has the same signs in all the variables involved in this loop while a balancing loop have opposite signs, since the intention is to reduce the effect of a certain variable to bring the system back to another (or the initial) desired state. A delay in the outcome is denoted by .

### Ethical considerations

Ethical approval for the four evaluations was obtained from the National Institute of Public Health’s Ethics and Research Commission. Key informants were told prior to the interviews about the objectives of the interview and the evaluation. Consent letters were signed by all those who accepted to be interviewed. All information provided by informants was safely stored in a computer where only the general coordinator of the evaluation had access to.

## Results

**Fig. 1:**
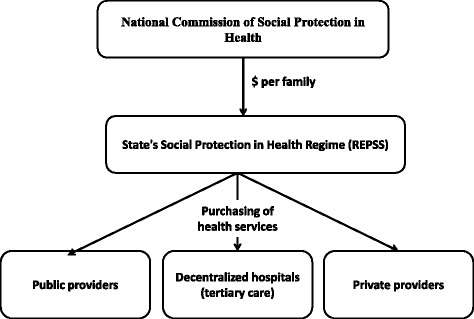
Initial design of *Seguro Popular* policy

In this section, we first describe the range of the main actors that were involved in the implementation of the policy, the characteristics of their interactions, and how they coped and adapted in reaction to the policy (Table 1). We then focus on how their responses led to modifications of the policy implementation over time followed by a description of the CAS phenomena that emerged from the analysis (Fig. 1).

### The range of actors and their responses to the policy

Table 1 describes the range of main actors involved in the operation of SPS by 2012, their incentives, role, power, and response to the policy implementation. The new role of main actors and how they emerged are described in turn below.

All actors identified are public and private actors that normally interact in the managerial processes that are carried out at federal and state levels. However, within SPS’s original design, some were not included, others were included and maintained their original role while a third group were included but modified their role.

The state’s treasuries were not explicitly mentioned in the original regulatory framework but they were the recipients of federal funds coming from the National Commission in order to register them to be subjected to federal audits. The assumption was that it would guarantee the transparent use of funds and facilitate accountability. In practice, the transfer of funds from treasuries to REPSS was seriously delayed (taking weeks and even months to be completed) which resulted in a new regulatory requirement for compulsory reports about delays [46]. Other federal health subsidies were by the State Secretariat of Health in a single pot to facilitate the distribution across the different programs that in some states created a great administrative confusion. REPSS in most states was located within the SSA’s structure and became subjected to the SSA decisions on how to pool and when to distribute the funds.

The participation of national workers union was not considered in the original design. It formally represents the interests of the SSA permanent workers but not of temporary employees. The union became agile to participate in negotiations to provide better labor conditions for the new short-term non-permanent employees that escalated after the introduction of SP [42, 47]. The negotiations resulted in setting a minimum period for short-term contracts of 12 months, with benefits included, and it provided the union with a 2 % salary contribution for each new contract as a union fee [42, 47]. These new conditions are known as the “regularization of contracts.”

The other important actor showing an emerging role is the pharmaceutical wholesale retailers. These enterprises, enjoying a limited market competition, also put pressure on the states SSA middle-range managers to influence purchasing decisions. Although the medicines purchasing process is carried out through public bidding, enterprises initially managed to negotiate excessively high prices in some states or provided cheaper alternatives to the products included in their bids after the signing of the contracts [28]. A number of pharmaceutical distributors identified a market niche in the transportation of medicines from SSA central premises to health care units, particularly those located in remote areas.

Finally, an actor that is diffused into the institutional structure is the SSA middle-range bureaucracy, which involves the various administrative levels all the way from the state secretariat to the front-line health providers. Being responsible for various expenditure decisions across the implementation process, several heterogeneous behavior and un-authorized expenses were reported such as purchasing of non-authorized vehicles and equipment, accepting bids for medicines well beyond the unitary prices defined by the federal government authorities, and contracting health workers that do not fulfil the minimum required skills or qualifications that receive salaries well above the norms [43]. While these practices have been detected by the federal audit system, the original SPS design did not foresee a penalty or sanction system that can allow the National Commission, responsible for tracking the use of funds, to address these practices in a legal way so while they could point it out to the respective administration, they had no power to enforce a change.

### Adaptations in the course of the policy implementation

**Fig. 2:**
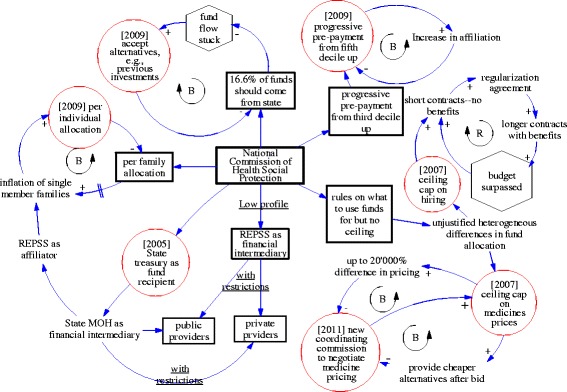
Causal loop diagrams illustrating the feedback loops and modifications of the policy

Figure 2 is a causal loop diagram that illustrates seven modifications or adaptations of the policy implementation (highlighted in circles) and the associated balancing and reinforcing feedback loops that evoked the changes. The figure should be read from the center outwards. In the center of the figure is the National Commission. The rectangles show the five main components of the policy identified and how each of them has been modified during the course of the implementation resulting in seven modifications (including six feedback loops) representing either the system’s or the actors’ response to the original design and how they coped with it or adapted it. Feedback loops were generated during the 10 years of implementation contributing to its modifications and adjustments, each one of them of a different nature. We discuss the seven modifications and the associated feedback loops in turn below, referring to the circles in Fig. 2 starting in a clockwise manner from the bottom.

Establishment of the State Secretariat of Health as de facto financial intermediary, rather than REPSS. Given that most REPSS were housed in the state’s SSA, the latter influenced all REPSS decisions and became the de facto financial allocator therefore halting the original intention to separate purchasing from provision of services [41, 42]. Not surprisingly, preference was given to allocate funds in public units and purchasing services from private providers was limited. In addition, historical budgeting rather than per capita budgeting remained the main criteria for allocation of funds to health units. This was attributed to a lack of managerial capacity within the state to switch to per capita budgeting and planning, limited information, and perceived complexity of setting up a whole new system to identify where population resided and what services are they effectively demanding and consuming.

*From family allocation to per capita allocation of funds*. In the original design, the capitation estimate was based on an average family size of 3.8 members. Funds started flowing based on these criteria in the initial years, but by 2007, the National Commission noted that the number of one-person families had grown unexpectedly in several states. According to the original guidelines, it was possible for above 18 individuals to be considered a new family unit even if they still reside with their parents which created an incentive to do so to increase the funds allocated to the various states. In response, the National Commission revised the operational guidelines in 2009 to base the per capita allocation of funds per capita and not per family unit. This revision benefited the Southern states, where poor families tend to be bigger in size [43].

*Changes in what the states’ financial contribution to SP’s fund is interpreted.* Each state was expected to provide proof of the availability of their state contribution up-front before receiving the remaining portion from the federal level. Several states struggled to demonstrate ear-marking these funds upfront, arguing that they were part of their ongoing investments to support the various health services for their population and therefore historic investments that have been incurred should be considered as part of their contribution. This dispute threatened the flow of funds to the states, which would hamper the impact of the policy as a whole. With more states supporting the arguments, the National Commission decided to develop new guidelines in 2009 that formalized this practice, therefore, guaranteeing the flow of federal resources through the system [43].

*Changes in pre-payment regulations.* The fourth source of funds for *Seguro Popular de Salud* is the pre-payment made by households belonging to the third income decile or higher. However, it became extremely difficult to collect pre-payment fees from households. Over the existence of SP around 92–95 % of the enrolled families did not pay fees [48]. After refining the instrument for identification of socio-economic status for the rest of income deciles and failing to increase fee collection, the National Commission opted to exempt the third and fourth income deciles from paying the fee, which was also formalized in 2009. The objectives of this change were mainly two: to be able to speed up the process of enrolment in order to attain the 100 % affiliation goal by 2012, the last year of the federal administration, and to reduce the costs of collecting the fee from those groups of populations that presented more difficulties to do so.

*Introducing a ceiling for expenditures on human resources.* Contracting of new health workers was the major source of expenditure in SP. In the early years, the National Commission noted that some states spent up to 70 % of the funds in contracting. In response, they negotiated a ceiling with the states for a maximum of 40 % of the total funds to be spent on this budget line. However, by 2010, after the change in contracting modality, 23 states were surpassing the 40 % ceiling which explains why this component still represents a major challenge [28, 41, 43].

*Introducing a ceiling for expenditures on medicines.* The second biggest source of expenditures has been purchasing and procurement of medicines. Similar to contracting, the National Commission negotiated with the states on establishing a ceiling of 30 % of total funds for the purchasing of medicines [28, 41, 43].

*Establishment of a new Coordinating Commission for the Negotiation of Prices of Medicines and other Inputs.* While medicines’ expenditures seemed to be contained within the 30 % ceiling, various coping strategies have emerged from pharmaceutical retailers in response to the cap. For example, by negotiating different unit prices for some medicines in different states and/or providing cheaper alternatives after the bid was accepted [18]. The National Commission, having noted this increasingly heterogeneous and irregular use of funds, convened key federal stakeholders and regulators together to establish new guidelines for the purchasing of medicines at national level. This involved the creation of a new Coordinating Commission for Negotiating the Price of Medicines and other Health Inputs that was responsible to negotiate, as a single public entity, unitary prices of medicines with individual drug manufacturers [49].

### Complex adaptive systems phenomena emerging from this analysis

In this section, we describe the various complex adaptive systems (CAS) phenomena that we identified through our analysis. They include phase transitions, unexpected consequences, resistance to change, history dependence, coping mechanisms, emergence of networks, delays and non-linear outcomes, and feedback loops.

Regarding phase transitions, the design of *Seguro Popular* represented a major transformation in the operational rationale of Mexico’s public health sub-system including innovations in financing, allocation of resources, definition of explicit packages of services, management procedures, and role and rights of users as newly “insured” populations. However, our analysis shows that the transition is partial and has mainly occurred in some of the components, particularly the financial component of the original design, expecting changes in other components to happen in further stages.

There are several examples of unexpected consequences that emerged from our analysis. One of them is the rise of the number of affiliated families, which was the basis for fund allocation to the states, due to the unexpected increase of one-person families. This example offers an interesting observation of how policies may be interpreted by its implementers when the details are not clearly defined or provide room for various interpretations. As the guidelines opened the possibility for the states to consider individuals above 18, who are not students, to be regarded a new family unit, probably assuming they may be in the workforce, several states interpreted this as a blank provision and therefore inflated the number of one-person families to increase the resource flows allocated to them.

Resistance to change typically appear in reaction to new policies and our analysis of *Seguro Popular de Salud* provides some examples. The first is the resistance of the state’s SSA to guarantee their share of resources to SPS as liquid funds before federal funds are disbursed. Another example is how households found ways to avoid paying the pre-payment conditional on income level. A third example is the strong preference of local health managers to maintain historical budgeting as the basis for allocating funds, rather than per capita based on new affiliates. This same example is also an illustration of history dependence where the past dictates the future and changing the ways of working and spending is not always easy or fast to implement.

Delays and non-linearity were reflected in the way various outcomes, and changes were observed in the various states. For example, the speed by which states started inflating the number of families and their strategies to negotiate the allocation of their 16.6 % co-responsibility as liquid funds up-front before the remaining of the fund is disbursed to them. The main non-linearity example is that the availability of resources (e.g., medicines, workers) and the capacity to produce services, particularly at the first level of care, does not correspond with the investment that *Seguro Popular de Salud* has made into the system. As to coping mechanisms, a good example is the alternatives pharmaceutical retailers’ sought in response to the imposed cap on expenditures on medicines as explained above.

Feedback loops occurred in various ways as described above and illustrated in the CLD in Fig. 2. Finally, various examples of emergence of networks and their collective impact were identified. For example, in the way how pharmaceutical distributors emerged and coalesced to develop a structured way of interacting with the implementers of the policy; how service providers collectively formed strong negotiation mechanisms to ensure that the *status quo* of historical funding prevails [23, 24]; and how the states gradually managed to exert pressure on the National Commission leading to their reformulation of the state’s contribution and what it entails.

## Discussion

One of the main strengths of this study is that it sought to apply a complexity lens through a CAS framework to understand the complex nature of implementing *Seguro Popular de Salud* in Mexico. While previous studies focused on top level parameters and indicators such as the increased volume and flow of funds to the health sector [13, 17, 18, 50], this study’s main interest is how did this happen and what modifications and coping mechanisms were involved, from the perspective of complex and adaptive systems. A limitation, however, is that it heavily relies on secondary data sources corresponding to a specific period of time (2007–2012). Thus, the current situation of SPS implementation could have changed from the one that was originally described.

The lessons of this analysis are applicable to any other settings aiming to incorporate a flow of fresh funds to improve the system’s performance and capacity. Our study emphasizes that no matter how evidence-based or logical the aim and design of a policy is, its implementation will undoubtedly follow different paths that are mostly unpredictable and unanticipated [51, 52]. Strategic governance of the systems and continuous evaluation and refining of policies are, therefore, essential for the execution of policies as it requires adequate knowledge of the complex behavior of health systems and substantial capacity of negotiation and leadership to be able to detect and adjust to the change in behavior of the system and its actors striving for maintaining the desired outcomes and goals and mitigating undesired consequences [53].

For example, resistance to change was also shown in other contexts has been associated with the implementation of new policies including financial reforms. In Tanzania, a financial reform that was aimed at expanding the health insurance coverage to the whole population failed to expand beyond 10 % of the population after 10 years of its implementation due to resistance from district level implementers who felt not involved in the design of the policy and that it was imposed on them from the central level [54]. History dependence in our case illustrated by the strong push by the local health managers to maintain historical budget level as the basis for allocating funds was also observed in other settings, e.g., in the Chinese reform,, emphasizing that changing the ways of working and spending is not always easy or fast to implement [55].

Two specific lessons for future financial reforms also emerged from our study. First, while Mexico attempted to avoid the risk of channelling the new financial flows through the private sector as in the case of Colombia in its 1990s reform, which created an enormous concentration of financial resources and corruption practices in these units [56], maintaining these new financial flows in the public sector exposed them to the power and incentives of the state governments which also have has a different set of risks and challenges as described in this paper. A second lesson is the difficulty of implementing a solidarity approach where the rich support the poor in collecting fees insurance premiums. In SPS, beneficiaries, including the rich, systematically avoided paying these fees, presumably because they did not see the benefits to them of doing so. The same experience was encountered in the Chinese reform [57].

In summary, applying a CAS framework to this analysis expanded our capacity to describe and understand this complex policy in a much more insightful and realistic ways, providing much richer and meaningful interpretation of the effects of the policy and a better understanding of how and why it was adapted in the course of its implementation. Policies that involve a monetary component share similar “risks” for perverse behaviors and this study carry lessons for relevant policies in other settings.

## Conclusions

Applying a systems thinking approach to future policy design, implementation, and evaluation, recognizing the characteristics of complex adaptive systems and embracing approaches that are consistent with this complexity would offer valuable steps forwards in the way policies are conceptualized, designed, and implemented. This process would involve the wider range of key stakeholders in the policy design stage, analyzing the incentives, roles and power of the key actors, including those that may emerge during the implementation of the policy, considering middle-range and front-line implementers as well as the beneficiaries, and brainstorming on the possible unexpected consequences with these various key actors to try and mitigate some of them [29].

## Abbreviations

CAS: complex adaptive systems
CJR: Clara Juárez-Ramírez
CLD: causal loop diagrams
GN: Gustavo Nigenda
IDRC: International Development Research Centre
LMGR: Luz María González-Robledo
NIPH: National Institute of Public Health of Mexico
PRI: Partido Revolucionario Institucional
REPSS: State’s Social Health Protection Regime
SP: Mexico’s *Seguro Popular*
SSA: Ministry of Health
SSoH: State’s Secretariats of Health
TA: Taghreed Adam
